# Parameter Estimation in Stratified Cluster Sampling under Randomized Response Models for Sensitive Question Survey

**DOI:** 10.1371/journal.pone.0148267

**Published:** 2016-02-17

**Authors:** Xiangke Pu, Ge Gao, Yubo Fan, Mian Wang

**Affiliations:** 1 Institute of Hepatology, Changzhou Third People’s Hospital, Changzhou, Jiangsu Province, 213001, P.R. China; 2 School of Public Health, Medical College of Soochow University, Suzhou, Jiangsu Province, 215123, P.R. China; 3 School of Public Health, Central South University, Changsha, Hunan Province, 410078, P.R. China; University of California, Berkeley, UNITED STATES

## Abstract

Randomized response is a research method to get accurate answers to sensitive questions in structured sample survey. Simple random sampling is widely used in surveys of sensitive questions but hard to apply on large targeted populations. On the other side, more sophisticated sampling regimes and corresponding formulas are seldom employed to sensitive question surveys. In this work, we developed a series of formulas for parameter estimation in cluster sampling and stratified cluster sampling under two kinds of randomized response models by using classic sampling theories and total probability formulas. The performances of the sampling methods and formulas in the survey of premarital sex and cheating on exams at Soochow University were also provided. The reliability of the survey methods and formulas for sensitive question survey was found to be high.

## Introduction

In surveys, researchers sometimes ask sensitive questions such as sex, income and drug abuse. Respondents often try to avoid answering sensitive questions because they are concerned with privacy. Some people will refuse to cooperate with researchers or give wrong answers to these questions. This will result in response bias and reporting errors in surveys on sensitive topics [1]. The randomized response technique was developed by Warner to collect sensitive information [2]. Privacy was argued to be maintained by the method so that response bias could be potentially removed. After that, modified randomized response models were proposed for different kinds of sensitive question survey. For example, Simmons model was introduced in dichotomous sensitive question survey [3][4]. The additive model was designed for quantitative sensitive question survey, which had advantages of simple design, small sample size, and reduced sampling error [5][6].

Simple random sampling has been considered a useful sampling method under randomized response models for sensitive question survey [7]. It seems to be easy to understand and apply by using random number table. However, this method is susceptible to sampling error if the sample is neither large nor representative. On the other hand, it is also cumbersome and costly when sampling from a large target population [8][9]. Compared to simple random sampling, other sophisticated sampling methods may be more suitable for a large population. For instance, stratified sampling uses prior information about the total population to get the sampling procedure more efficient and it reduces sampling error, while cluster sampling has the advantages of less cost and convenience by separating the population into clusters [10][11]. But these methods are not widely used in sensitive question survey because they are not easy to understand like simple random sampling. So formulas for simple random sampling may be incorrectly used instead of a relatively complex but efficient sampling procedure in sensitive question survey. Even if effective sampling has been performed, the reliability of sampling methods under randomized response models has seldom been evaluated [12][13].

In this work, we provided designs for cluster sampling and stratified cluster sampling under two kinds of randomized response models. Corresponding formulas for parameter estimation were also deduced. These complex sampling methods have been successfully employed in survey of premarital sex and cheating at Soochow University and may be more applicable to a large population.

## Survey Methods

### Dichotomous sensitive question survey

#### Simmons model on dichotomous sensitive question survey

In Simmons model, a randomized device is designed like that some red balls and some white balls are mixed together and put into a bag. Before responding, a respondent randomly takes a ball out of the bag, identifies the color of the ball and then puts it back. When the red ball is drawn, he/she would give a yes or no answer to a question with sensitive character A, while the white ball is taken out, he/she would also give a yes or no answer to the other question with non-sensitive character B. These balls are the same except for color and the red ball’s probability of being selected should not be 50% [5].

#### Cluster sampling under Simmons model

It is convenient to use cluster sampling method in science research. The cluster sampling process in Simmons model is as follows: 1). The population is divided into several clusters (primary units), and each cluster is composed of secondary units. 2). Some clusters are randomly selected from the population. 3). Simmons model (1.1.1) is applied to all the secondary units of selected clusters for dichotomous sensitive question survey.

#### Stratified cluster sampling under Simmons model

Stratified sampling is useful in reducing sampling error. Based on cluster sampling, the stratified cluster sampling process in Simmons model is as follows: 1). The population is stratified into different strata by characters; 2). Each stratum is divided into different clusters (primary units) respectively; 3). Each cluster is composed of secondary units; 4). Simmons model (1.1.1) is employed to all the secondary units to conduct dichotomous sensitive question survey.

### Quantitative sensitive question survey

#### The additive model on quantitative sensitive question

In the additive model [5], a randomized device is designed to randomly generate an integer between 0 and 9. In the device, ten same balls are respectively labeled by the ten integers. By a randomization process, each respondent takes an integer-labeled ball and add the integer to the numerical value of his response to the sensitive question to get a final result.

#### Cluster sampling under the additive model on quantitative sensitive question

Cluster sampling (1.1.2) is used and the additive model is applied to subunits for quantitative sensitive question survey.

#### Stratified cluster sampling under the additive model on quantitative sensitive question

Stratified cluster sampling (1.1.3) is used and the additive model is employed to secondary units for quantitative sensitive question survey.

## Formula Deductions

### Estimation of the population proportion for sensitive question survey

#### Formulas for cluster sampling

Suppose the population is divided into *N* clusters, and the *i*th cluster contains *Mi* subunits, and then *n* clusters are drawn from the population randomly.

Estimation of the population proportion and its variance is described below:

Suppose the proportion of sensitive character A in the *i*th cluster is *π*_*i*_, and the *i*th cluster contains *a*_*i*_ secondary units with character A (*π*_*i*_ = *a*_*i*_ / *M*_*i*_), and the proportion of character A in the population is π.

**①** When each cluster contains *M* secondary units, the estimator of π is[8]

$$\hat{\pi }=\frac{1}{nM}\sum_{i=1}^{n}a_{i}=\frac{1}{n}\sum_{i=1}^{n}\frac{a_{i}}{M}=\frac{1}{n}\sum_{i=1}^{n}\pi_{i}$$ (1)

The estimator of variance of $\hat{\pi }$ can be stated as [8]

$$v(\hat{\pi })=\frac{1-f}{n\left(n-1\right)}\sum_{i=1}^{n}{\left(\pi_{i}-\hat{\pi }\right)}^{2},$$ (2)

where *f* = *nM* / *NM* = *n* / *N* is the sampling ratio.

**②** When all the clusters are not the same size, we can approximately get the estimator of π

$$\hat{\pi }=\frac{\sum_{i=1}^{n}a_{i}}{\sum_{i=1}^{n}M_{i}}$$ (3)

The estimator of the variance of $\hat{\pi }$ can be obtained as

$$v(\hat{\pi })=\frac{1-f}{n{\bar{M}}^{2}}\frac{\sum_{i=1}^{n}a_{i}^{2}-2\hat{\pi }\sum_{i=1}^{n}a_{i}M_{i}+{\hat{\pi }}^{2}\sum_{i=1}^{n}M_{i}^{2}}{n-1}$$ (4)

where $\bar{M}=\sum_{i=1}^{n}M_{i}/n$ is the mean of the subunits in each cluster, $f=\frac{\sum_{i=1}^{n}M_{i}}{\sum_{i=1}^{N}M_{i}}$ is the sampling ratio.

From the equation *π*_*i*_ = *a*_*i*_ / *M*_*i*_, we can get

$$v(\hat{\pi })=\frac{1-f}{n{\bar{M}}^{2}}\frac{\sum_{i=1}^{n}M_{i}^{2}{\left(\pi_{i}-\hat{\pi }\right)}^{2}}{n-1}$$ (5)

Calculation of *π*_*i*_ and *a*_*i*_ is described below:

Let *P* denote the proportion of the sensitive question in the randomized device of the Simmons model, and the proportion of people with non-sensitive character B in the *i*th cluster is *R*_*i*_, which is known or can be obtained. *λ*_*i*_ denotes the proportion of a “yes” response in the *i*th cluster and *π*_*i*_ denotes the proportion of character A in the *i*th cluster. From full probability formulas [14], we can get

$$\lambda_{i}=\pi_{i}\cdot P+\left(1-P\right)R_{i},$$

So we have

$$\pi_{i}=\frac{\lambda_{i}-(1-P)R_{i}}{P}$$ (6)

and

$$a_{i}=M_{i}\pi_{i}\;,i=1,2,\ldots ,n$$

#### Formulas for stratified cluster sampling

Suppose the population is composed of *L* strata, and the *h*th stratum contains *N*_*k*_ clusters (primary units), and the *i*th cluster contains *M*_*ih*_ secondary units. The population contains *N* secondary units and *n*_*h*_ clusters are randomly drawn from the *h*th stratum.

Estimation of the population proportion in the *h*th stratum and its variance is described below:

Suppose *π*_*ih*_ denotes the proportion of subunits with sensitive character A in the *i*th cluster of the *h*th stratum, and there are *a*_*ih*_ subunits with character A.

**①** When each cluster in the *h*th stratum contains *M*_*h*_ subunits (clusters in different strata can be of different size), and from formula 1, the estimator of population proportion *π*_*h*_ for cluster sampling in the *h*th stratum is

$${\hat{\pi }}_{h}=\frac{1}{n_{h}}\sum_{i=1}^{n_{h}}\pi_{ih}\;,h=1,2,\ldots ,L$$ (7)

From (2), the estimator of the variance of ${\hat{\pi }}_{h}$ (the estimator of $V({\hat{\pi }}_{h})$) can be stated as

$$v({\hat{\pi }}_{h})=\frac{1-f_{h}}{n_{h}\left(n_{h}-1\right)}\sum_{i=1}^{n_{h}}{\left(\pi_{ih}-{\hat{\pi }}_{h}\right)}^{2}\;,h=1,2,\ldots ,L$$ (8)

where *f*_*h*_ = *n*_*h*_*M*_*h*_ / *N*_*h*_*M*_*h*_ = *n*_*h*_ / *N*_*h*_ is the sampling ratio of the *h*th stratum.

**②** When all the clusters are not the same size, from formula 3, the estimator of the population proportion in the *h*th stratum can be obtained as

$${\hat{\pi }}_{h}=\frac{\sum_{i=1}^{n_{h}}a_{ih}}{\sum_{i=1}^{n_{h}}M_{ih}}\;,h=1,\;2\;,\ldots ,\;L$$ (9)

And from formula 4, the estimator of the variance of ${\hat{\pi }}_{h}$ can be got as

$$v({\hat{\pi }}_{h})=\frac{1-f_{h}}{n_{h}{\bar{M}}_{h}{}^{2}}\frac{\sum_{i=1}^{n_{h}}a_{ih}^{2}-2{\hat{\pi }}_{h}\sum_{i=1}^{n_{h}}a_{ih}M_{ih}+{\hat{\pi }}_{h}{}^{2}\sum_{i=1}^{n_{h}}M_{ih}^{2}}{n_{h}-1}\;,\;h=1,\;2\;,\ldots ,\;L$$ (10)

and by formula 5, we can get

$$v({\hat{\pi }}_{h})=\frac{1-f_{h}}{n_{h}{{\bar{M}}_{h}}^{2}}\frac{\sum_{i=1}^{n_{h}}M_{ih}^{2}{\left(\pi_{ih}-{\hat{\pi }}_{h}\right)}^{2}}{n_{h}-1}\;,\;h=1,\;2\;,\ldots ,\;L$$ (11)

where ${\bar{M}}_{h}=\sum_{i=1}^{n_{h}}M_{ih}/n_{h}$ is the mean of subunits of each cluster in the *h*th stratum,

and $f_{h}=\frac{\sum_{i=1}^{n_{h}}M_{ih}}{\sum_{i=1}^{N_{h}}M_{ih}}$ is the sampling ratio in the *h*th stratum.

Estimation of the population proportion and its variance is described below:

The estimator of the population proportion can be stated as [8]

$$\hat{\pi }=\frac{\sum_{h=1}^{L}\sum_{i=1}^{N_{h}}M_{ih}{\hat{\pi }}_{h}}{N}=\sum_{h=1}^{L}W_{h}{\hat{\pi }}_{h}$$ (12)

where $W_{h}=\sum_{i=1}^{N_{h}}M_{ih}/N$ is the relative size of the *h*th stratum according to the number of subunits.

As the sample of each stratum is relatively independent, from formula 12 we can get the variance of ${\hat{\pi }}_{h}$ [15]

$$V(\hat{\pi })=\sum_{h=1}^{L}W_{h}^{2}V({\hat{\pi }}_{h})$$ (13)

According to the size of each cluster of the *h*th stratum, we can use formula 8, 9 and 11 to estimate the $V({\hat{\pi }}_{h})$ in formula 13.

Calculation of *π*_*ih*_ and *a*_*ih*_ is described below:

Suppose the proportion of subunits with unrelated non-sensitive character B in the *i*th cluster of the *h*th stratum is *R*_*ih*_. *R*_*ih*_ is known or can be acquired by special survey. *λ*_*ih*_ denotes the proportion of a “yes” answer in the *i*th cluster of the *h*th stratum and *π*_*ih*_ denotes the proportion of character A in the *i*th cluster. From full probability formula [14], we can get

$$\pi_{ih}=\frac{\lambda_{ih}-\left(1-P\right)R_{ih}}{P}\;h=1,2,\;\ldots \;,\;L;\;i=1,2,\ldots \;,n_{\;h}$$ (14)

and *a*_*ih*_ = *M*_*ih*_*π*_*ih*_, *i* = 1,2,…,*n*_*h*_*; h =* 1,2,…,*L*

### Estimation of the population mean for sensitive question survey

#### Formulas for cluster sampling

Estimation of the population mean and its variance is described below:

**①** When *M*_*i*_ = *M*, let *μ*_*i*_ denote the mean of the *i*th cluster, and replacing *a*_*i*_ by *y*_*i*_ in formula 1, the estimator of the population mean can be stated as

$$\hat{\mu }=\frac{1}{nM}\sum_{i=1}^{n}y_{i}=\frac{1}{n}\sum_{i=1}^{n}\frac{y_{i}}{M}=\frac{1}{n}\sum_{i=1}^{n}\mu_{i}\;,i=1,2,\;\ldots \;,\;n$$ (15)

When *a*_*i*_ is also replaced by *y*_*i*_ in formula 2, the estimator of the variance of $\hat{\mu }$ can be obtained as

$$v(\hat{\mu })=\frac{1-f}{n\left(n-1\right)}\sum_{i=1}^{n}{\left(\mu_{i}-\hat{\mu }\right)}^{2}$$ (16)

where *f* = *nM* / *NM* = *n* / *N* is the sampling ratio.

**②** When all the clusters are not the same size, the estimator of the population mean is [16]

$$\hat{\mu }=\frac{\sum_{i=1}^{n}y_{i}}{\sum_{i=1}^{n}M_{i}}$$ (17)

By replacing *a*_*i*_ by *y*_*i*_ in formula 4, the estimator of the variance of $\hat{\mu }$ can also be got as [8]

$$v(\hat{\mu })=\frac{1-f}{n{\bar{M}}^{2}}\frac{\sum_{i=1}^{n}y_{i}^{2}-2\hat{\mu }\sum_{i=1}^{n}y_{i}M_{i}+{\hat{\mu }}^{2}\sum_{i=1}^{n}M_{i}^{2}}{n-1}$$ (18)

where $\bar{M}=\sum_{i=1}^{n}M_{i}/n$ is the mean of the subunits in each cluster, and $f=\frac{\sum_{i=1}^{n}M_{i}}{\sum_{i=1}^{N}M_{i}}$ is the sampling ratio.

When $\hat{\pi }$ is replaced by $\hat{\mu }$ in formula 5, we can get

$$v(\hat{\mu })=\frac{1-f}{n{\bar{M}}^{2}}\frac{\sum_{i=1}^{n}M_{i}^{2}{\left(\mu_{i}-\hat{\mu }\right)}^{2}}{n-1}$$ (19)

Calculation of *μ*_*i*_ and *y*_*i*_ is described below:

Suppose *μ*_*i*_ denotes the mean of variables with sensitive character in the *i*th cluster, and *μ*_iZ_ denotes the mean of numerical values of the answers in the *i*th cluster, and *μ*_*Y*_ denotes the mean of all the random numbers in the randomized device. And then from characteristics of means [15], we can get

$$\mu_{\text{iZ}}=\mu_{\text{i}}+\mu_{Y}\;,\;i=1,2,\ldots ,n$$

So

$$\mu_{i}=\mu_{iZ}-\mu_{Y}\;,\;i=1,2,\ldots ,n$$ (20)

And we can get

$$y_{i}=M_{i}\mu_{i}\;,\;i=1,2,\ldots \;,n$$

#### Formulas for stratified cluster sampling

Estimation of the population mean and its variance of the *h*th stratum is described below:

**①** When *M*_*ih*_ = *M*_*h*_, from formula 15, we can get the estimator of the population mean

$${\hat{\mu }}_{h}=\frac{1}{n_{h}M_{h}}\sum_{i=1}^{n_{h}}y_{ih}=\frac{1}{n_{h}}\sum_{i=1}^{n_{h}}\frac{y_{ih}}{M_{h}}=\frac{1}{n_{h}}\sum_{i=1}^{n_{h}}\mu_{ih}\;,\;h=1,2,\ldots ,L$$ (21)

And from formula 16, the estimator of the variance of ${\hat{\mu }}_{h}$ can be obtained as

$$v({\hat{\mu }}_{h})=\frac{1-f_{h}}{n_{h}\left(n_{h}-1\right)}\sum_{i=1}^{n_{h}}{\left(\mu_{ih}-{\hat{\mu }}_{h}\right)}^{2}\;,\;h=1,2,\ldots ,L$$ (22)

**②** When all the clusters in the *h*th stratum are not the same size, from formula 17, the estimator of the population mean (*μ*_*h*_) of the *h*th stratum is

$${\hat{\mu }}_{h}=\frac{\sum_{i=1}^{n_{h}}y_{ih}}{\sum_{i=1}^{n_{h}}M_{ih}}\;,\;h=1,2,\ldots ,L$$ (23)

From formula 18, the estimator of the variance of ${\hat{\mu }}_{h}$ can be stated as

$$v({\hat{\mu }}_{h})=\frac{1-f}{n_{h}{\bar{M}}_{h}^{2}}\frac{\sum_{i=1}^{n}y_{ih}^{2}-2{\hat{\mu }}_{h}\sum_{i=1}^{n_{h}}y_{ih}M_{ih}+{\hat{\mu }}_{h}^{2}\sum_{i=1}^{n_{2}}M_{ih}^{2}}{n_{h}-1}\;,\;h=1,2,\ldots ,L$$ (24)

And from formula 19, we can get a simplified formula

$$v({\hat{\mu }}_{h})=\frac{1-f_{h}}{n_{h}{\bar{M}}_{h}^{2}}\frac{\sum_{i=1}^{n_{h}}M_{ih}^{2}{\left(\mu_{ih}-{\hat{\mu }}_{h}\right)}^{2}}{n_{h}-1}\;,\;h=1,2,\ldots ,L$$ (25)

Estimation of the population mean and its variance is described below:

Through replacing ${\hat{\pi }}_{h}$ by ${\hat{\mu }}_{h}$ in formula 12, we can get the estimator of the population mean[8]:

$$\hat{\mu }=\frac{\sum_{h=1}^{L}\sum_{i=1}^{N_{h}}M_{ih}{\hat{\mu }}_{h}}{N}=\sum_{h=1}^{L}W_{h}{\hat{\mu }}_{h}$$ (26)

As samples of each stratum are independent, by formula 26, we can get

$$V(\hat{\mu })=\sum_{h=1}^{L}W_{h}^{2}V({\hat{\mu }}_{h})$$ (27)

According to the size of the clusters of the *h*th stratum and by formula 22, 24 and 25, we can estimate $V({\hat{\mu }}_{h})$ in formula 27.

Calculation of *μ*_*ih*_ and *y*_*ih*_ is described below:

Let *μ*_ih_ denote the mean of variables with sensitive character in the *i*th cluster of the *h*th stratum, *μ*_iZ_ denote the average value of all the answers in the *i*th cluster of the *h*th stratum, and *μ*_*Y*_ denote the average value of all the random numbers in the randomized device. Then from characteristics of means [15], we can get

$$\mu_{\text{ihZ}}=\mu_{\text{ih}}+\mu_{Y}$$

Thus,

$$\mu_{ih}=\mu_{ihZ}-\mu_{Y}\;,i=1,2,\cdots ,\;{\text{n}}_{\text{h}};h=1,2,\cdots ,L$$ (28)

And then we can get

$$y_{ih}=M_{ih}\mu_{ih}$$

### Applications

Let the students on Dushu Lake Campus of Soochow University be the target population which is divided into two strata. Define undergraduates as the first stratum which contains 9689 students and graduates as the second stratum which contains 1890 students, we can get *W*_1_ = 9689/(9689 + 1890) ≈ 0.84, *W*_2_ ≈ 0.16. Let each class to be a cluster and clusters in each stratum to be approximately the same size, 20 clusters which contain 1080 students were randomly drawn from undergraduates and 18 clusters containing 818 students were drawn from graduates. Each student was repeatedly surveyed twice at different time and 3796 times of survey were conducted in total. All the questionnaires were recovered and the passing rate of the questionnaires was 100%. Data bank was established using Excel 2003 and all the data was analyzed by SAS 9.13.

### Survey on a population proportion

In our randomized device, there were 6 red balls and 4 white balls with the same size and weight in a bag. In the absence of others, each student chose a ball from the bag randomly. When the red ball was drawn, he should answer the sensitive question that whether he had premarital sex. And when the white ball was chosen, he should answer the unrelated question that whether he was a boy. The student could only give a yes or no answer and finally the real proportion (*R*_*ih*_) of boys in each cluster of each stratum should be acquired.

#### Ethics Statement

The participants provided their written informed consent to participate in this study anonymously. The data were also collected and analyzed anonymously. This research was approved by the Ethics Committee of Soochow University including the consent procedure.

#### The proportion of students having premarital sex in each class

Survey on premarital sex of students from 38 classes at Soochow University was repeated twice by stratified cluster sampling under Simmons model. From formula 14, we can get the premarital sex rate *π*_*i*1_ ((*i* = 1,2,…,20) in the first survey of undergraduates and $\pi_{i1}^{\prime }$ ((*i* = 1,2,…,20) in the second survey of undergraduates, and the premarital sex rate *π*_*i*2_ (*i* = 1,2,…,18) in the first survey of graduates and $\pi_{i2}^{\prime }$ ((*i* = 1,2,…,18) in the second survey of graduates (Table 1).

**Table 1:** Results of survey on premarital sex of students from 38 classes by stratified cluster sampling in the Simmons model.

Serial numbers of undergraduate classes	*π*_*i*1_	$\pi_{i1}^{\prime }$	Serial numbers of graduate classes	*π*_*i*2_	$\pi_{i2}^{\prime }$
1	0.2624	0.2270	1	0.2348	0.2348
2	0.1631	0.1277	2	0.2296	0.1926
3	0.2101	0.2464	3	0.2403	0.2403
4	0.2063	0.2460	4	0.2148	0.2522
5	0.1556	0.1926	5	0.2041	0.2624
6	0.2390	0.2151	6	0.2000	0.2333
7	0.1783	0.2171	7	0.2270	0.2624
8	0.1970	0.1970	8	0.2270	0.1915
9	0.0476	0.0079	9	0.2296	0.2667
10	0.1224	0.0884	10	0.2639	0.2292
11	0.0455	0.0076	11	0.2411	0.2766
12	0.1185	0.1185	12	0.2292	0.2639
13	0.0903	0.1273	13	0.2340	0.1986
14	0.2727	0.2348	14	0.2381	0.1984
15	0.1884	0.1884	15	0.4222	0.3852
16	0.1439	0.1439	16	0.2264	0.2264
17	0.1746	0.1349	17	0.2803	0.2045
18	0.1594	0.1957	18	0.2803	0.2202
19	0.1984	0.1587			
20	0.1926	0.1556			

#### Estimation of premarital sex proportion and its variance in each stratum

By the first survey and formula 7, we can get the estimator of the premarital sex ratio of undergraduates:

$${\hat{\pi }}_{1}=\frac{1}{n_{1}}\sum_{i=1}^{n_{1}}\pi_{i1}=\frac{1}{20}\times \left(0.2624+0.1631+\cdots +0.1926\right)=0.1683$$

From formula 8, we can get the estimator of the variance of ${\hat{\pi }}_{1}$:

$$\begin{array}{l} v({\hat{\pi }}_{1})=\frac{1-f_{1}}{n_{1}\left(n_{1}-1\right)}\sum_{i=1}^{n_{1}}{\left(\pi_{i1}-{\hat{\pi }}_{1}\right)}^{2}\\ =\frac{1-1080/9689}{20\left(20-1\right)}\left[{\left(0.2624-0.1683\right)}^{2}+{\left(0.1631-0.1683\right)}^{2}+\cdots +{\left(0.1926-0.1683\right)}^{2}\right]\\ =0.0002 \end{array}$$

By the first survey and formula 7, we can also get the estimator of the premarital sex ratio of graduates:

$${\hat{\pi }}_{2}=\frac{1}{n_{2}}\sum_{i=1}^{n_{2}}\pi_{i2}=\frac{1}{18}\times \left(0.2348+0.2296\;+\cdots +0.2803\right)=0.2457$$

And from formula 8, we can get the estimator of the variance of ${\hat{\pi }}_{2}$:

$$\begin{array}{l} v({\hat{\pi }}_{2})=\frac{1-f_{2}}{n_{2}\left(n_{2}-1\right)}\sum_{i=1}^{n_{2}}{\left(\pi_{i2}-{\hat{\pi }}_{2}\right)}^{2}\\ =\frac{1-818/1890}{18\left(18-1\right)}\left[{\left(0.2348-0.2457\;\right)}^{2}+{\left(0.22296-0.2457\;\right)}^{2}+\cdots +{\left(0.2803\;-0.2457\;\right)}^{2}\right]\\ =0.0001 \end{array}$$

#### Estimation of the premarital sex proportion and its variance of the students on Dushu Lake Campus of Soochow University

By formula 12, we can get the estimator of the premarital sex ratio of the students:

$$\hat{\pi }=\sum_{h=1}^{L}W_{h}\cdot {\hat{\pi }}_{h}=W_{1}{\hat{\pi }}_{1}+W_{2}{\hat{\pi }}_{2}=0.84\times 0.1683+0.16\times 0.2457=0.1807$$

And from formula 13, we can get the estimator of the variance of $\hat{\pi }$:

$$\begin{array}{l} v(\hat{\pi })=\sum_{h=1}^{L}W_{h}^{2}\cdot v({\hat{\pi }}_{h})=W_{1}^{2}\cdot v\left({\hat{\pi }}_{1}\right)+W_{2}^{2}\cdot v\left({\hat{\pi }}_{2}\right)\\ =0.84^{2}\times 0.0002+0.16^{2}\times 0.0001=0.0001 \end{array}$$

Thus, the 95% confidence interval of the population proportion is given by

$$\hat{\pi }\pm 1.96\times \sqrt{v(\hat{\pi })}=0.1954\pm 1.96\times \sqrt{0.0001}=0.1758∼0.2150$$

#### Reliability evaluation

By using SAS 9.13, the data from the repeat survey of the 38 classes were carried out arcsine transformation of square root. The correlative analysis showed that the results of the two repeated sample surveys were coincident and the reliability of our survey methods and formulas was high (coefficient of product-moment correlation r = 0.8843, P<0.0001).

### Survey on a population mean

In our randomized device, there were 10 balls of identical size in a bag and the balls were respectively tagged by integers from 0 to 9. Each chosen student was asked to select a ball from the bag and get a corresponding integer, and added the number to times of his cheating on exams in last two semesters and wrote down the final result.

#### The mean of times of cheating on exams in each class

Through twice repeated surveys on cheating on exams in last two semesters by stratified cluster sampling under the additive model and by formula 28, we can get the average times of cheating on exams of undergraduates in 20 classes in the first survey (*μ*_*i*1_ (*i* = 1,2,…,20)) and that in the second survey ($\mu_{i1}^{'}$ (*i* = 1,2,…,20)), and the mean of times of cheating on exams of graduates in 18 classes in the first survey (*μ*_*i*2_ (*i* = 1,2,…,18)), and that in the second survey ($\mu_{i2}^{'}$ (*i* = 1,2,…,18)) can also be got (Table 2).

**Table 2:** Results of survey on cheating of students in 38 classes by stratified cluster sampling under the additive model.

Serial numbers of undergraduate classes	*μ*_*i*1_	$\mu_{i1}^{'}$	Serial numbers of graduate classes	*μ*_*i*2_	$\mu_{i2}^{'}$
1	1.0532	0.9894	1	1.3182	1.4318
2	2.0250	2.3500	2	2.4302	2.7791
3	1.6000	1.5000	3	2.6556	2.4778
4	1.6702	1.8617	4	1.1170	1.4149
5	1.0682	1.1134	5	0.9694	0.9490
6	0.6500	0.5930	6	0.9200	0.9200
7	1.1667	1.0111	7	0.8556	1.0556
8	1.2021	1.0745	8	0.5652	0.8404
9	1.1667	0.7444	9	0.5667	0.5444
10	1.1122	1.1531	10	0.7708	0.7500
11	1.2143	1.4524	11	0.7766	0.7553
12	0.5227	0.5000	12	0.7500	0.7917
13	1.1000	1.0111	13	0.7553	0.7340
14	0.7955	0.7727	14	0.5238	0.5000
15	0.9348	0.9130	15	1.0333	1.0556
16	0.9318	0.8636	16	0.9340	0.8874
17	0.1522	0.1087	17	0.7955	0.7727
18	0.9524	0.8810	18	0.9000	0.9181
19	0.8333	0.7857			
20	0.5222	0.5000			

#### Estimation of the population mean and its variance of cheating times in each stratum

Estimation of the population mean of cheating times in each stratum is described below:

By the first survey on undergraduates and formula 21, we can get the estimator of the population mean of cheating times of undergraduates in last two semesters:

$${\hat{\mu }}_{1}=\frac{1}{20}(1.0532\;+2.0250+\cdots +0.5222)=1.0337$$

By the first survey on graduates and formula 21, we can also get the estimator of the population mean of cheating times of graduates in last two semesters:

$${\hat{\mu }}_{2}=\frac{1}{18}(1.3182+2.4302+\cdots +0.9000)=1.0354$$

Estimation of the variance of the population mean of cheating times in each stratum is described below:

From the first survey on undergraduates and formula 22, we can get the estimator of the variance of the population mean of cheating times of undergraduates in last two semesters:

$$\begin{array}{l} v({\hat{\mu }}_{1})=\frac{1-f_{1}}{n_{1}\left(n_{1}-1\right)}\sum_{i=1}^{n_{1}}{\left(\mu_{i1}-{\hat{\mu }}_{1}\right)}^{2}\\ =\frac{1-1080/9689}{20\left(20-1\right)}\left[{\left(1.0532-1.0337\right)}^{2}+{\left(2.0250-1.0337\right)}^{2}+\cdots +{\left(0.5222-1.0337\right)}^{2}\right]\\ =0.0079 \end{array}$$

From the first survey on graduates and formula 22, we can also get the estimator of the variance of the population mean of cheating times of graduates in last two semesters:

$$\begin{array}{l} v({\hat{\mu }}_{2})=\frac{1-f_{2}}{n_{2}\left(n_{2}-1\right)}\sum_{i=1}^{n_{2}}{\left(\mu_{i2}-{\hat{\mu }}_{2}\right)}^{2}\\ =\frac{1-818/1890}{18\left(18-1\right)}\left[{\left(1.3182-1.0354\;\right)}^{2}+{\left(2.4302-1.0354\right)}^{2}+\cdots +{\left(0.9000-1.0354\right)}^{2}\right]\\ =0.0107 \end{array}$$

#### Estimation of the population mean and its variance of cheating times of students on Dushu Lake Campus of Soochow University

By formula 26, we can get the estimator of the population mean of cheating times of students on Dushu Lake Campus of Soochow University:

$$\hat{\mu }=\sum_{h=1}^{2}W_{h}{\hat{\mu }}_{h}=0.84\times 1.0337+0.16\times 1.0354=1.0340$$

By formula 22 and 27, we can get the estimator of the variance of the population mean of cheating times of students on Dushu Lake Campus of Soochow University:

$$v(\hat{\mu })=\sum_{h=1}^{2}W_{h}^{2}v({\hat{\mu }}_{h})=0.84^{2}\times 0.0079+0.16^{2}\times 0.0107=0.0058$$

Thus, the 95% confidence interval of the population mean of cheating times of students on Dushu Lake Campus of Soochow University is given by

$$\hat{\mu }\pm 1.96\sqrt{\nu \left(\hat{\mu }\right)}=1.0340\pm 1.96\sqrt{0.0058}=0.8847∼1.1833$$

#### Reliability evaluation

Correlative analysis was applied to the data of two repeated surveys under the additive model in 20 undergraduate classes by using SAS 9.13. The Shapiro-Wilks’ W test was applied to *μ*_*i*1_ and $\mu_{i1}^{'}$ and the corresponding values of W were 0.9616 and 0.9288 respectively; the P values were 0.5768 and 0.1464 respectively, and were normally distributed. This analysis showed that the coincidence between results of the two repeated cluster samplings in the first stratum was high (coefficient of product-moment correlation r = 0.95755, P<0.0001). Rank correlation analysis was applied to the data of two repeated surveys under the additive model in 18 graduate classes by using SAS 9.13 and also showed that the coincidence was high (the Spearman rank correlation coefficient *r*_*s*_ = 0.90243, P<0.0001, not normal distribution). Rank correlation analysis was also applied to the data of all the 38 classes and the results of the two repeated stratified cluster samplings was verified to be coincident (the Spearman rank correlation coefficient *r*_*s*_ = 0.90311,P<0.0001, not normal distribution), which indicate that our survey methods and relevant formulas were reliable.

## Discussion

Sensitive question survey is very important in social and medical research, especially for the prevention of AIDS in China. After going through the introduction period and the growth period of AIDS, China is now facing the threat of AIDS outbreak. To prevent AIDS, accurate data are needed although many people may be unwilling to give true answers to such a sensitive question. Survey methods and formulas for parameter estimation proposed in this study will be helpful to get reliable data to prevent sexually transmitted diseases and improve public health.

Randomized response models have been widely used to make people cooperative in sensitive question survey. Recently a Meta-analysis was applied to 38 relevant publications from 1965 to 2000 and showed that the application of randomized response models has a significant advantage of accuracy and reliability compared with other traditional survey methods [17]. As to the sampling design for sensitive question survey, statisticians have provided a lot of sampling methods. However, most cases have been limited to simple random sampling so far, and studies on the evaluation of the reliability and validity of sample survey on sensitive questions are also rare.

In this work, formulas for parameter estimation in cluster sampling and stratified cluster sampling under two randomized response models were deduced respectively and both dichotomous and quantitative data about sensitive issues could be acquired. Under the two models, cluster sampling and stratified cluster sampling were successfully applied to the survey of premarital sex and cheating at Soochow University. The evaluation of the test-retest reliability demonstrated that the coincidence of results between two repeated surveys was high and our survey methods and statistical formulas are highly reliable.

As for cluster and stratified cluster sampling, the sample size is generally large. So the sample proportion and sample mean usually follow the normal distribution. With the deduced formulas, we can get estimators of all kinds of population proportions, means and their variances; we can estimate the intervals of those population proportions and means; and we can further compare the proportion and mean of each stratum by t-test, Z test, analysis of variance or rank test.

Multistage sampling, cluster sampling, stratified cluster sampling and other complex sampling methods and corresponding formulas under randomized response models for survey of quantitative and qualitative sensitive issues are under study in the project. In this study, cluster sampling and stratified cluster sampling methods and relevant formulas under two randomized response models are proved to be reliable and will probably be used for sensitive question survey of a large population.

## References

[1] Roger T, Ting Y (2007) Sensitive questions in surveys. Psychol Bull 133: 859–883. 17723033 10.1037/0033-2909.133.5.859

[2] Warner SL (1965) Randomized response: A survey technique for eliminating answer bias. J Am Stat Assoc 60: 63–69. 12261830

[3] Horvitz DG, Shah BV, Simmons WR. (1967) The unrelated question randomized response model. Proceedings of Social Stat Sec Am Stat Assoc: 65–72.

[4] Greenberg BG, Abul-Ela AA, Simmons WR, Horvitz DG. (1969) The unrelated question randomized response: theoretical framework. J Am Stat Assoc 64: 520–539.

[5] Wang J (2003) Practical Medical Research Methods Beijing: People’s Medical Publishing House. 420–450 p.

[6] Raghunath A, Georg D (2006) Randomized response techniques for complex survey designs. Stat Pap 48: 131–141.

[7] Sun S (2004) Sampling survey Beijing: Peking University Press. 177–189 p.

[8] Cochran WG (1977) Sampling techniques (3rd ed.). New York: John Wiley & Sons. 280–289 p.

[9] Guo X (2005) Practical medical survey techniques Beijing: People’s Military Medical Press. 39–40 p.

[10] Meng B, Yu H, Zheng L, Yao G. (2012) A comparative research via sampling methods: A case study of the commute time in Beijing. Journal of Beijing Union University 26: 10–16.

[11] Ding Y, Gao G (2008) Health Statistics Beijing: Science Press. 13–20 p.

[12] Lv X, Liu Y (2011) Comparative study on two situation of additive model under simple random sampling with replacement. Journal of Inner Mongolia Agricultural University (Natural Science Edition) 32: 313–317.

[13] Liu W, Gao G, Li X. (2010) Stratified random sampling on simmons model for sensitive question survey. Suzhou University Journal Of Medical Science 30: 759–762,776.

[14] Su L (2007) Advanced Mathematical Statistics Beijing: Peking University Press. 3 p.

[15] Wang Y, Sui S, Wang A (2006) Mathematical Statistics and engineering data analysis by MATLAB Beijing: Tsinghua University Press. 442–450 p.

[16] Wang J, Gao G, Fan Y, Chen L, Liu S, Jin Y, et al (2006) The estimation of sampling size in multi-stage sampling and its application in medical survey. Applied Mathematics and Computation 178: 239–249.

[17] Lensvelt-Mulders GJLM, Hox JJ, van der Heijden PGM, Maas CJM (2005) Meta-analysis of randomized response research, thirty-five years of validation. Sociol Methods Res 33: 319–348.

